# The effect of probiotic supplementation on the gut microbiota of preterm infants

**DOI:** 10.1099/jmm.0.001403

**Published:** 2021-08-25

**Authors:** Erica L. Plummer, Jennifer A. Danielewski, Suzanne M. Garland, Jenny Su, Susan E. Jacobs, Gerald L. Murray

**Affiliations:** ^1^​ Centre for Women’s Infectious Diseases Research, The Royal Women’s Hospital, Parkville, Victoria, Australia; ^2^​ Infection & Immunity Theme, Murdoch Children’s Research Institute, Melbourne, Victoria, Australia; ^3^​ Central Clinical School, Monash University, Melbourne, VIC, Australia; ^4^​ Department of Obstetrics and Gynaecology, University of Melbourne, Parkville, Victoria, Australia; ^5^​ Neonatal Services, The Royal Women’s Hospital, Parkville, Victoria, Australia; ^6^​ Clinical Sciences, Murdoch Children’s Research Institute, Melbourne, Victoria, Australia

**Keywords:** *Bifidobacterium longum *subsp. *infantis*, *Bifidobacterium animalis* subsp. *lactis*, gut microbiota, preterm infants, probiotics, *Streptococcus thermophilus*

## Abstract

**Introduction:**

Probiotic supplementation of preterm infants may prevent serious morbidities associated with prematurity.

**Aim:**

To investigate the impact of probiotic supplementation on the gut microbiota and determine factors associated with detection of probiotic species in the infant gut.

**Hypothesis/Gap Statement:**

Probiotic supplementation increases the long-term colonization of probiotic species in the gut of preterm infants.

**Methodology:**

Longitudinal stool samples were collected from a cohort of very preterm infants participating in a blinded randomized controlled trial investigating the impact of probiotic supplementation (containing *Bifidobacterium longum* subsp. *infantis* BB-02, *Bifidobacterium animalis* subsp. *lactis* BB-12 and *Streptococcus thermophilus* TH-4) for prevention of late-onset sepsis. The presence of *B. longum* subsp. *infantis *, *B. animalis* subsp. *lactis* and *S. thermophilus* was determined for up to 23 months after supplementation ended using real-time PCR. Logistic regression was used to investigate the impact of probiotic supplementation on the presence of each species.

**Results:**

Detection of *B. longum* subsp. *infantis* [odds ratio (OR): 53.1; 95 % confidence interval (CI): 35.6–79.1; *P* < 0.001]*, B. animalis subsp. *lactis** (OR: 89.1; 95 % CI: 59.0–134.5; *P* < 0.001) and *S. thermophilus* (OR: 5.66; 95 % CI: 4.35–7.37; *P* < 0.001) was increased during the supplementation period in infants receiving probiotic supplementation. Post-supplementation, probiotic-supplemented infants had increased detection of *B. longum* subsp. *infantis* (OR: 2.53; 95 % CI: 1.64–3.90; *P* < 0.001) and *B. animalis* subsp. *lactis* (OR: 1.59; 95 % CI: 1.05–2.41; *P*=0.030). Commencing probiotic supplementation before 5 days from birth was associated with increased detection of the probiotic species over the study period (* B. longum* subsp. infantis*,* OR: 1.20; *B. animalis* subsp. lactis*,* OR: 1.28; *S. thermophilus,* OR: 1.45).

**Conclusion:**

Probiotic supplementation with *B. longum* subsp. *infantis* BB-02, *B. animalis* subsp. *lactis* BB-12 and *S. thermophilus* TH-4 enhances the presence of probiotic species in the gut microbiota of very preterm infants during and after supplementation. Commencing probiotic supplementation shortly after birth may be important for improving the long-term colonization of probiotic species.

## Introduction

Preterm infants have an altered gastrointestinal microbiota compared with term infants, including reduced species diversity, reduced numbers of *Bifidobacterium* species and *Lactobacillus* species, and higher numbers of potentially pathogenic *Enterobacteriaceae* and *Clostridium difficile* [1, 2]. These differences probably result from reduced exposure to maternal microbiota, from immaturity of the intestinal immune response, and from the various medical interventions necessary in management within neonatal intensive care units [3].

Probiotics are defined by the World Health Organisation as ‘live microorganisms which when administered in adequate amounts confer a health benefit on the host’ [4]. Recent systematic reviews and meta-analyses have reported that probiotic supplementation is effective for the prevention of serious morbidities associated with prematurity, including mortality, late-onset sepsis (LOS) and necrotizing enterocolitis (NEC) [5–8]. The *ProPrems* randomized controlled trial (RCT) was a large multi-centre double blind, placebo-controlled randomized trial investigating the effect of supplementation with a probiotic combination (* Bifidobacterium longum* subsp. *infantis* strain BB-02, *Streptococcus thermophilus* strain TH-4 and *Bifidobacterium animalis* subsp. *lactis* strain BB-12*,* containing a total of 1×10^9^ organisms) on LOS in very preterm infants [9, 10]. A secondary objective of the *ProPrems* RCT was to investigate the impact of the probiotic combination on the incidence of NEC. The *ProPrems* RCT found that while administration of the probiotic was not associated with reduction in LOS or mortality, it resulted in a 54 % reduced risk of NEC of Bell stage 2 or more [10].

The exact mechanism(s) by which probiotics exert benefits in preterm infants is not known and may be strain-specific. One likely mechanism is modulation of gut microbiota composition [11]. We previously reported findings from a 16S rRNA gene pyrosequencing study of 66 infants who participated in the *ProPrems* RCT [12]. We found that infants who received probiotic were more likely to have increased abundance of *Bifidobacterium* in their gut microbiota during the supplementation period compared to infants randomized to placebo; however, no difference in the abundance of *Streptococcus* was observed [12]. This study was limited in that it only provided genus-level resolution and thus could not identify if the elevated bifidiobacteria were subspecies found in the probiotic combination. In the present study, we investigated the impact of probiotic supplementation on detection of *B. longum* subsp. *infantis *, *B. animalis* subsp. *lactis* and *S. thermophilus* in the gut microbiota of a subset of *ProPrems* infants using real-time PCR (qPCR). We also determined concordance of the 16S rRNA gene sequencing data [12] and qPCR for the detection of *Bifidobacterium *.

## Methods

### Specimen collection and laboratory methods

The *ProPrems* RCT protocol and details on specimen collection have been reported elsewhere [9, 10, 12]. Briefly, preterm infants born weighing less than 1500 g and less than 32 weeks of gestation were randomized to receive either a probiotic combination at 1.5 g per day [1×10^9^ total organisms of *B. longum* subsp. *infantis* BB-02 (300×10^6^), *S. thermophilus* TH-4 (350×10^6^) and *B. animalis* subsp. *lactis* BB-12 (350×10^6^; ABC Dophilus Probiotic Powder for Infants; Solgar), in a maltodextrin base powder] or placebo (maltodextrin powder). Infants commenced the assigned intervention when they were tolerating at least 1 ml of milk feeds every 4 h. Infants continued to receive the intervention until discharge home or term-corrected age, whichever came first. Stool swabs were collected from Victorian infants as close to the following time points as possible: prior to commencement of the study powder, after 1, 4 and 8 weeks of study powder, and at 6 and 12 months of age corrected for prematurity. An anal swab was collected if the infant had not yet passed a stool, and a swab of the first meconium was collected where available.

Swabs were rotated in 400 µl of PBS and extracted using the MagNA Pure 96 nucleic acid extraction system (Roche Diagnostics) and the MagNA Pure 96 DNA and viral small volume Isolation Kit, as previously described [13]. Extracted DNA was tested using qPCR assays targeting the 16S rRNA gene region for the detection of *B. longum* subsp. *infantis *, *B. animalis* subsp. *lactis *, *S. thermophilus* [9, 13], and all *Bifidobacterium* targeting the 16S–23S rRNA intergenic spacer region [14] (Table 1). PCR and cycling conditions were as previously outlined (95 °C for 10 min followed by 50 cycles of 95 °C for 10 s and 60 °C for 55 s) [13]. PCR cycling conditions for all four qPCR assays were the same, allowing 22 samples (plus one negative and one positive control per assay) to be amplified for all four targets in one run on the LightCycler 480 real-time instrument (Roche Diagnostics). Samples were analysed in four subsets of 24 (a subset for each assay) using the second derivative maximum method to obtain the quantification cycle (Cq) for each target. As a positive control, 1.55 g of ABC Dophilus Probiotic Powder for Infants was resuspended in 3.62 ml PBS, from which 200 µl was extracted as described [13]. A serial dilution of the calculated copies per organism was performed, and the dilution estimated at 1000 copies per 5 µl was used as a positive control. The methodology for sample preparation for 454 pyrosequencing (using a Roche 454 Genome Sequencer instrument; GS FLX Titanium Chemistry), sequence processing and taxonomic assignment has been previously reported [12]. Briefly, gut microbiota composition was determined for 215 specimens using bifidobacteria-optimized PCR primers [15] that amplify the V3–V5 hypervariable regions of the 16S rRNA gene. Sequencing data were processed with QIIME (Version 1.8.0) [16], using UCLUST (Version 1.2.22q) [17] to cluster sequences into operational taxonomic units using a similarity threshold of 97 %. The UCLUST consensus taxonomy assigner (Version 1.2.22q) [17] was used to assign taxonomy using default parameters and the silva reference database (Version 111) [18].

**Table 1.** Primers and probes used in this study

Target	Primer sequence (5′−3′)*	Reference
* B. longum* subsp. *infantis *	F: TTCCAGTTGATCGCATGGTC	[31]
	R: GGAAACCCCATCTCTGGGAT	
	P: CY5-TCAAgCCCAggTAAggTTCTTCgC BHQ3	[13]
* B. animalis* subsp. *lactis *	F: GTGGAGACACGGTTTCCC	[32]
	R†: CACACCACACAATCCAATAC	
	P: FAM-TTCACAGGTGGTGCATGGTCGT BHQ1	[13]
* S. thermophilus *	F: TTATTTGAaAGgGGCaATTGCT	[13, 27] Modified
	R: GTGAACttTCCACTCtCACAC	
	P: CY5-ACTACAAGATGGACCTGCGT BHQ3	
All *Bifidobacterium *	F†: GGGATGCTGGTGTGGAAGAGA	[14]
	R†: TGCTCGCGTCCACTATCCAGT	
	P†: FAM-TCAAACCACCACGCGCCA BHQ1	

*Lower case letters indicate locked nucleic acid (LNA) bases.

†Primers/probe is located within the 16S–23S rRNA intergenic spacer region; all other primers and probes are located in the 16S rRNA gene region, which is more highly conserved.

F, forward primer; R, reverse primer; P, probe; FAM, 6-carboxyfluorescein; Cy5, cyanine 5; BHQ, Black Hole Quencher.

### Statistical analysis

The qPCR data were initially expressed as a binary variable, i.e. detected (Cq ≤ 45) or not detected (Cq > 45). Logistic regression models that accounted for repeated measures within infants were fitted using generalized estimating equations (GEEs) to investigate the association between probiotic supplementation and detection of *B. longum* subsp. infantis*, B. animalis subsp. *lactis** and *S. thermophilus* in the gut microbiota. We grouped specimens into three categories based on timing of collection and investigated the association between probiotic supplementation and detection of *B. longum* subsp. infantis*, B. animalis subsp. *lactis** and *S. thermophilus* in the gut microbiota before, during and after supplementation. Logistic regression was also used to determine what factors were associated with detection of probiotic species in infants randomized to the probiotic group.

Bacterial abundance of *B. longum* subsp. infantis*, B. animalis subsp. *lactis** and *S. thermophilus* was approximated using Cq values and summarized using box plots. A low Cq value indicates a higher copy number of the target organism than high Cq values. Specimens with no detectable target were assigned a Cq value of 45. Differences in Cq values between probiotic and placebo infants at the three time points (i.e. before, during and after supplementation) were assessed using linear regression models fitted using GEEs.

Sequencing data for *Bifidobacterium* were expressed as a binary variable. Specimens were classified as detected if they had any sequence reads assigned to *Bifidobacterium* and not detected if there were no sequence reads assigned. Concordance of *Bifidobacterium* detection using 16S rRNA gene sequencing and *Bifidobacterium* detected using the genus-specific qPCR assay was determined using Cohen’s kappa statistic.

All analyses were carried out using STATA (Version 14; StataCorp).

## Results

A total of 683 infants were recruited to the *ProPrems* trial at hospitals located in Victoria, Australia. Specimens were collected from 680 infants and qPCR data were available for 472 infants. Thirteen infants and their specimens were excluded because they did not receive the allocated intervention. As a result, 459 infants contributed 2335 specimens to this analysis. This included 1181 specimens from 229 infants randomized to the probiotic combination and 1154 specimens from 230 infants randomized to the placebo. A median of five specimens [interquartile range (IQR): 4–6] were collected from infants. Participant characteristics are summarized in Table 2. Two-thirds of infants were born by Caesarean section (68 %) and 28 infants developed NEC during the study. One infant developed NEC at 5 months of age after their participation in the study ended. Characteristics were similar between the allocation groups, except that more infants were born at ≤ 28 weeks of gestation in the placebo group [47, 95 % confidence interval (CI): 40–53 %] compared to the probiotic group (35 %, 95 % CI: 39–42 %).

**Table 2.** Participant characteristics

Characteristic	qPCR sub-study participants (*N*=459)	Probiotic (*N*=229)	Placebo (*N*=230)
Gestational age, weeks, median (IQR)	28.3 (27–30)	28.6 (27.2–30)	28.1 (26.5–29.5)
< 28 weeks, *n* (%)			
No	271 (59)	148 (65)	123 (53)
Yes	188 (41)	81 (35)	107 (47)
Birth weight, g, median (IQR)	1058 (854–1275)	1080 (875–1284)	1045 (845–1262)
< 1000 g, *n* (%)			
No	258 (56)	131 (57)	127 (55)
Yes	201 (44)	98 (43)	103 (45)
Male, *n* (%)			
No	231 (50)	114 (50)	117 (51)
Yes	228 (50)	115 (50)	113 (49)
Caesarean delivery, *n* (%)			
No	145 (32)	70 (31)	75 (33)
Yes	314 (68)	159 (69)	155 (67)
Any NEC, *n* (%)			
No	430 (94)	218 (95)	212 (92)
Yes	29 (6)	11 (5)	18 (8)
Age commenced study powder, days, median (IQR)	5 (4–7)	5 (4–7)	5 (4–7)
Length of supplementation, days, median (IQR)	64 (47–79)	63 (48–78)	65 (47–80)
Specimens included, *n*, median (IQR)	5 (4–6)	5 (4–6)	5 (4–6)
Number of specimens, number of infants			
Before supplementation	679, 447	341, 225	338, 222
During supplementation	1251, 446	630, 223	621, 223
Post-supplementation	401, 259	207, 132	194, 127

IQR, interquartile range; NEC, necrotizing enterocolitis.

Over the whole study period, infants randomized to probiotic were more likely to have *B. animalis* subsp. *lactis* [odds ratio (OR): 10.2; 95 % CI: 8.35–12.5; *P* < 0.001, Table 3], *B. longum* subsp. *infantis* (OR: 7.16; 95 % CI: 5.89–8.69; *P* < 0.001) and *S. thermophilus* (OR: 2.64; 95 % CI: 2.25–3.11; *P* < 0.001) detected than infants randomized to placebo.

**Table 3.** Effect of probiotic supplementation on detection of probiotic species before, during and after supplementation

	Probiotic		Placebo		OR (95 % CI)*	*P* value
	No. of infants(%)†	No. of specimens (%)‡	No. of infants(%)†	No. of specimens (%)‡		
Over whole study period	***n***=229	***n***=1178	***n***=230	***n***=1153		
* B. animalis* subsp. *lactis *	222 (97)	666 (57)	89 (39)	128 (11)	10.2 (8.35–12.5)	**<0.001**
* B. longum* subsp. *infantis *	225 (98)	729 (62)	112 (49)	214 (19)	7.16 (5.89–8.69)	**<0.001**
* S. thermophilus *	216 (95)	611 (52)	171 (75)	333 (29)	2.64 (2.25–3.11)	**<0.001**
**Before supplementation**	***n*=225**	***n*=341**	***n*=222**	***n*=338**		
* B. animalis* subsp. *lactis *	8 (4)	8 (2)	3 (1)	3 (1)	2.67 (0.72–9.99)	0.144
* B. longum* subsp. *infantis *	13 (6)	13 (4)	8 (4)	8 (2)	1.62 (0.67–3.88)	0.282
* S. thermophilus *	15 (7)	15 (4)	11 (5)	13 (4)	1.17 (0.52–2.62)	0.700
**During supplementation**	***n*=223**	***n*=630**	***n*=223**	***n*=621**		
* B. animalis* subsp. *lactis *	220 (99)	553 (88)	39 (17)	48 (8)	89.1 (59.0–134.5)	**<0.001**
* B. longum* subsp. *infantis *	222 (99)	582 (93)	82 (37)	123 (20)	53.1 (35.6–79.4)	**<0.001**
* S. thermophilus *	205 (92)	418 (66)	114 (51)	161 (26)	5.66 (4.35–7.37)	**<0.001**
**After supplementation**	***n*=132**	***n*=207**	***n*=127**	***n*=194**		
* B. animalis* subsp. *lactis *	84 (64)	105 (51)	63 (49)	77 (40)	1.59 (1.05–2.41)	**0.030**
* B. longum* subsp. *infantis *	103 (78)	134 (65)	66 (52)	83 (43)	2.53 (1.64–3.90)	**<0.001**
* S. thermophilus *	120 (90)	178 (86)	112 (88)	159 (82)	1.40 (0.76–2.57)	0.283

*GEE logistic regression clustered for multiple specimens from each infant.

†Number and percentage of infants who provided at least one specimen in which the target was detected.

‡Number and percentage of specimens in which the target was detected.

A total of 679 specimens were collected prior to starting supplementation [median age of collection=2 days (range=0–15); Table S1, available in the online version of this article]. There was no significant difference in the detection of *B. longum* subsp. infantis*, B. animalis subsp. *lactis** and *S. thermophilus* before supplementation started between the probiotic and placebo groups (Table 3). A total of 1251 specimens were collected during the supplementation period (median age of collection=30 days, range=0–96 days; Table S1). Detection of *B. longum* subsp. *infantis* (OR: 53.1; 95 % CI: 35.6–79.4; *P* < 0.001)*, B. animalis subsp. *lactis** (OR: 89.1; 95 % CI: 59.0–134.5; *P* < 0.001) and *S. thermophilus* (OR: 5.66; 95 % CI: 4.35–7.37; *P* < 0.001) was increased during the supplementation period in infants receiving probiotic. A total of 401 specimens were collected post-supplementation [median age of collection=275 days (range=13–731); collected a range of 1 day to 23 months after ending supplementation], with the majority of post-supplementation specimens collected 6–12 months after the last dose of powder (*n*=203/401, 51 %; Table S1). Post-supplementation, infants who received probiotic had increased detection of *B. longum* subsp. *infantis* (OR: 2.53; 95 % CI: 1.64–3.90; *P*=0.030) and *B. animalis* subsp. *lactis* (OR: 1.59; 95 % CI: 1.05–2.41; *P* < 0.001), but no difference in detection of *S. thermophilus* (OR: 1.40; 95 % CI: 0.76–2.57; *P*=0.283).

We observed no difference in the Cq value of *B. longum* subsp. *infantis *, *B. animalis* subsp. *lactis* and *S. thermophilus* between randomization groups prior to supplementation (Fig. S1 and Table S2). During the supplementation period, the Cq value of the three probiotic species was lower (indicating higher abundance) in infants receiving probiotic compared to placebo infants [* B. longum* subsp. *infantis* median Cq value=22 (IQR: 19, 27) vs. 45 (IQR: 45, 45), coefficient=−16.63, 95 % CI: –17.68, –15.58; *P* < 0.001, *B. animalis* subsp. *lactis* median Cq value=32 (IQR: 28, 36) vs. 45 (IQR: 45, 45), coefficient=−11.70, 95 % CI: –12.29, –11.09; *P* < 0.001 and *S. thermophilus* median Cq value=45 (IQR: 40, 45) vs. 45 (IQR: 45, 45), coefficient=−1.41, 95 % CI: –1.92, –0.89; *P* < 0.001]. Post-supplementation we observed lower Cq values of *B. longum* subsp. *infantis* in probiotic-supplemented infants [median Cq value=31 (IQR: 23, 45) vs. 45 (IQR: 28, 45), coefficient=−4.69, 95 % CI: –6.77, –2.61; *P* < 0.001], but no difference in the Cq values of *B. animalis* subsp. *lactis* or *S. thermophilus *. While the Cq values of *B. longum* subsp. *infantis* and *B. animalis* subsp. *lactis* were lowest during the supplementation period, the Cq values of *S. thermophilus* were lowest post-supplementation.

Using logistic regression, we examined the association between key variables (including gestational age, birth weight, antibiotic exposure, age started supplementation and duration of supplementation) and the detection of the probiotic species over the whole study period in only those infants who received the probiotic (Table S3). Commencing probiotic supplementation within 5 days after birth was associated with increased detection of *B. animalis* subsp. *lactis* (OR: 1.20; 95 % CI: 1.02–1.41; *P*=0.033), *B. longum* subsp. *infantis* (OR: 1.28; 95 % CI: 1.09–1.49; *P*=0.002) and *S. thermophilus* (OR: 1.45; 95 % CI: 1.19–1.77; *P* < 0.001). Receiving 65 days or more of probiotic was associated with increased detection of *B. animalis* subsp. *lactis* (OR: 1.25; 95 % CI: 1.06–1.47; *P*=0.008), but not the other two species. Gestational age ≤ 28 weeks was associated with decreased detection of *S. thermophilus* (OR: 0.73; 95 % CI: 0.59–0.90; *P*=0.003), and low birth weight (< 1000 g) was associated with decreased detection of both *S. thermophilus* (OR: 0.79; 95 % CI: 0.64–0.96; *P*=0.020) and *B. longum* subsp. *infantis* (OR: 0.84; 95 % CI: 0.72–0.99; *P*=0.033). Infants exposed to antibiotics were less likely to have detection of the three probiotic species, but this was only statistically significant for *S. thermophilus* (OR: 0.76; 95 % CI: 0.62–0.93; *P*=0.007). Breastmilk feeding rates in this cohort were high (97 %) [10] so we could not investigate if breast feeding was associated with detection of probiotic species.

In total, 127 specimens had both 16S rRNA gene sequencing data and qPCR data available. There was substantial concordance for the detection of *Bifidobacterium* (91 %, kappa coefficient=0.79, *P* < 0.001, Table 4).

**Table 4.** Concordance of qPCR and 16S rRNA gene sequencing for detection of *Bifidobacterium
*

		qPCR*		
		Not detected	Detected	Row totals
16S rRNA gene sequencing†	Not detected	30	4	34
	Detected	7	86	93
	Column totals	37	90	127

Concordance 91 %, kappa coefficient=0.79, *P* <0.001.

*Genus-specific primers for *Bifidobacterium* targeting the 16S–23S rRNA intergenic spacer were used, and *Bifidobacterium* was considered detected by qPCR if Cq value <45.

†*Bifidobacterium* was considered detected by 16S rRNA gene sequencing if any sequence reads were assigned to the genus *Bifidobacterium*.

## Discussion

In this study we investigated the impact of probiotic supplementation with *B. longum* subsp. *infantis* BB-02, *S. thermophilus* TH-4 and *B. animalis* subsp. *lactis* BB-12 on the gut microbiota of a subset of infants who participated in the *ProPrems* RCT [9, 10]. Our results indicate that the probiotic combination increases the detection of *B. longum* subsp. infantis*, B. animalis subsp. *lactis** and *S. thermophilus* in the gut microbiota of preterm infants during supplementation. While detection of *B. longum* subsp. *infantis* and *B. animalis* subsp. *lactis* was increased post-supplementation in infants who received the probiotic combination, there was no difference in the detection of *S. thermophilus* post-supplementation between the probiotic and placebo groups (Table 3). These data extend the existing 16S rRNA gene sequencing data from this cohort [12] by providing subspecies-level resolution from a large number of infants. Importantly, our results suggest that commencing probiotics closer to birth may be important for ensuring long-term colonization.

Our findings are consistent with previous studies that report increased detection of probiotic strains in probiotic-supplemented infants compared to controls. A small longitudinal study investigating the gut microbiota of only seven infants supplemented with Infloran (a probiotic containing *B. bifidum* and *Lactobacillus acidophilus *) and three control infants found, using qPCR assays, that *B. bifidium* was increased in probiotic-supplemented infants during and after supplementation [19]. *L. acidophilus* was rarely detected in either probiotic or control infants [19]. The PiPs study, a large RCT (*n*=1310 preterm infants) of the probiotic *B. breve* BBG-001, found that 84 % of probiotic-supplemented infants were colonized with *B. breve* BBG-001 by strain-specific qPCR at 2 weeks of age compared to 35 % of control infants [20]. Additionally, using culture methods, *B. breve* was detected in the stool of 84 % of probiotic infants compared to 49 % of placebo infants at 36 weeks of postmenstrual age [20]. Using a species-specific qPCR assay, Patole *et al.* [21] found that *B. breve* was detected in 91 % (*n*=67/74) of infants supplemented with *B. breve* M-16V vs. 38 % (*n*=25/66) of control infants 3 weeks after supplementation.

The presence of the probiotic species during supplementation is not necessarily confirmation that colonization of these species has occurred: rather it may simply represent detection of ingested probiotic that has passed through the gastrointestinal tract. Infants in our study provided specimens for up to 23 months post-supplementation, with the majority of specimens collected 6–12 months after ending supplementation (Table S1). Increased detection of probiotic species post-supplementation as demonstrated in our study (for *B. longum* subsp. *infantis* and *B. animalis* subsp. *lactis *) and previous studies [19–22] suggests long-term colonization. *B. longum* subsp. *infantis* had a relatively lower Cq post-supplementation compared to *B. animalis* subsp. *lactis *, suggesting that *B. longum* subsp. *infantis* may be more effective at colonizing the gut. This is supported by a two-part study of 17 infants that reported increased faecal *Bifidobacterium* (by 16S rRNA gene sequencing) in infants supplemented with *B. longum* subsp. *infantis* compared to infants supplemented with *B. animalis* subsp. *lactis* [23], but this was a small study with limited samples collected post-supplementation.

There is limited information regarding the optimal time to start probiotics; however, our results suggest that commencing shortly after birth may be important for colonization (Table S3). In addition, we found that early gestational age (<28 weeks), low birth weight (<1000 g) and antibiotic use were each associated with decreased detection of one or more of the probiotic species. Consistent with this, the PiPs study reported that in infants randomized to probiotic, increasing gestational age was associated with increased colonization and antibiotic use was associated with decreased colonization [20]. Early gestational age, low birth weight and antibiotic use are known to influence the gut microbiota composition [24], and are characteristics of a higher risk group of premature infants. It is possible that the negative association between these factors and probiotic colonization is a result of the immaturity of the gastrointestinal tract in these at-risk infants. Importantly, antibiotic use has been shown to reduce the abundance of beneficial bacteria including *Bifidobacterium* species and increase the abundance of potentially pathogenic bacteria in preterm infants [25]. Thus it is possible that these alterations to the gut microbiota impede colonization of the probiotic species.

*S. thermophilus* was the most prevalent species detected post-supplementation and no difference in detection was observed between probiotic and placebo-supplemented infants (Table 3). Most specimens collected post-supplementation were collected at 6 and 12 months corrected age, when infants had probably commenced a solid food diet. *S. thermophilus* is commonly used in the production of yoghurt and some cheeses [26], so the frequent detection of *S. thermophilus* post-supplementation in both allocation groups may suggest it was acquired through diet.

There are limitations to this study. First, the qPCR assays used were not strain-specific. As a result, we cannot be certain that the *B. longum* subsp. *infantis *, *B. animalis* subsp. *lactis* and *S. thermophilus* detected were the probiotic strains. However, the increased detection of probiotic species in probiotic-supplemented infants compared to controls during the supplementation period suggests that the probiotic strains were present in these infants. Second, the PCR primers used for detection of *S. thermophilus* also detect *S. salivarius* [27]. A whole genome or metagenomic approach would provide higher specificity for detecting the probiotic strains and may reveal if genomic differences in *B. longum* subsp. *infantis *, *B. lactis* and *S. thermophilus* exist between probiotic and control infants. Finally, the detection of the probiotic species in samples collected prior to supplementation could indicate some cross-colonization took place while the infants were in the neonatal unit, which has previously been reported [20, 28–30].

## Conclusion

We report that probiotic supplementation with *B. longum* subsp. *infantis* BB-02, *S. thermophilus* TH-4 and *B. animalis* subsp. *lactis* BB-12 enhances the presence of *B. longum* subsp. infantis*, B. animalis subsp. *lactis** and *S. thermophilus* in the gut microbiota of preterm infants during supplementation. We followed infants for up to 23 months post-supplementation and found increased detection of both *Bifidobacterium* probiotic species in infants randomized to probiotic compared to placebo infants, which suggests that probiotic use may result in long-term colonization. Importantly, we report that commencing probiotic supplementation shortly after birth may be important for improved long-term colonization of probiotic species.

## Supplementary Data

Supplementary material 1
