# Supplementary material for: The effect of probiotic supplementation on the gut microbiota of preterm infants
Source: J Med Microbiol. 2021 Aug 25;70(8):001403. doi: 10.1099/jmm.0.001403 (PMC8513625; doi:10.1099/jmm.0.001403)
Supplement: Supplementary material 1 [file jmm-70-1403-s001.pdf]

Supporting Information

Supplementary Table 1

	Number of specimens collected n	Age in days when specimen collected Median (range)	Days since powder ended Median (range)
Before supplementation	679	2 (0-15)	
During supplementation	1251	30 (2-96)	
Post supplementation	401	275 (13-731)	199 (1-689)
Months since powder ended			
< 2 months	101	61 (13-99)	14 (1-58)
2-6 months	25	260 (147-290)	177 (86-182)
6-12 months	203	282 (234-473)	208 (183-365)
12-18 months	65	470 (114-613)	394 (366-484)
>18 months	7	685 (635-731)	591 (575-689)

Supplementary Table 2.

	Probiotic	Placebo	Coefficient (95% CI)[b]	P value
	Median Cq value[a] (IQR)	Median Cq value[a] (IQR)		
**Before supplementation**				
B. animalis subsp. *lactis*	45 (45,45)	45 (45,45)	-0.20 (-0.49,0.08)	0.163
B. longum subsp. *infantis*	45 (45,45)	45 (45,45)	-0.35 (-0.73,0.03)	0.069
S. thermophilus	45 (45,45)	45 (45,45)	0.12 (-0.19,0.42)	0.459
**During supplementation**				
B. animalis subsp. *lactis*	32 (28,36)	45 (45,45)	-11.70 (-12.29,-11.09)	**<0.001**
B. longum subsp. *infantis*	22 (19,27)	45 (45,45)	-16.63 (-17.68,-15.58)	**<0.001**
S. thermophilus	45 (40,45)	45 (45,45)	-1.41 (-1.92,-0.89)	**<0.001**
**After supplementation**				
B. animalis subsp. *lactis*	45 (34,45)	45 (34,45)	-0.83 (-2.24,0.59)	0.253
B. longum subsp. *infantis*	31 (23,45)	45 (28,45)	-4.69 (-6.77,-2.61)	**<0.001**
S. thermophilus	37 (33,42)	38 (33,44)	-0.24 (-1.50,1.02)	0.710

IQR, interquartile range; CI, confidence interval

[a] Cq refers to the quantification cycle, which was used as an approximation for bacterial abundance. A low Cq value indicates a higher copy number of the target organism than high Cq values.

[b] GEE linear regression clustered for multiple specimens from each infant

Supplementary Table 3

	B. animalis subsp. *lactis* (N=1178)				*B. longum* subsp. *infantis* (N=1177)				*S. thermophilus* (N=1178)			
	**Not detected**	**Detected**	**OR (95% CI)[a]**	**pvalue**	**Not detected**	**Detected**	**OR (95% CI)[a]**	**pvalue**	**Not detected**	**Detected**	**OR (95% CI)[a]**	**pvalue**
**<28 weeks GA**												
No	321 (42)	440 (58)	1		285 (38)	475 (62)	1		346 (45)	415 (55)	1	
Yes	191 (46)	226 (54)	0.86 (0.72-1.02)	0.085	163 (39)	254 (61)	0.90 (0.76-1.07)	0.223	221 (53)	196 (47)	0.73 (0.59-0.90)	**0.003**
**<1000g birthweight**												
No	285 (43)	374 (57)	1		241 (37)	417 (63)	1		300 (46)	359 (54)	1	
Yes	227 (44)	292 (56)	0.98 (0.83-1.16)	0.797	207 (40)	312 (60)	0.84 (0.72-0.99)	**0.033**	267 (51)	252 (49)	0.79 (0.64-0.96)	**0.020**
**Delivery mode**												
Vaginal	168 (46)	201 (54)	1		136 (37)	233 (63)	1		189 (51)	180 (49)	1	
Caesarean	344 (42)	465 (58)	1.08 (0.90-1.29)	0.392	312 (39)	496 (61)	0.90 (0.76-1.07)	0.241	378 (47)	431 (53)	1.17 (0.94-1.46)	0.154
**Antibiotic use**												
No	213 (42)	293 (58)	1		185 (37)	320 (63)	1		224 (44)	282 (56)	1	
Yes	299 (44)	373 (56)	0.90 (0.76-1.06)	0.216	263 (39)	409 (61)	0.88 (0.75-1.04)	0.132	343 (51)	329 (49)	0.76 (0.62-0.93)	**0.007**
**Commenced powder <5 days from birth**												
No	300 (46)	3659 (54)	1		265 (40)	394 (60)	1		344 (52)	315 (48)	1	
Yes	212 (41)	307 (59)	1.20 (1.02-1.41)	**0.033**	183 (35)	335 (65)	1.28 (1.09-1.49)	**0.002**	223 (43)	296 (57)	1.45 (1.19-1.77)	**<0.001**
**Length of supplementation**												
≤64 days	277 (47)	316 (53)	1		238 (40)	354 (60)	1		282 (48)	311 (52)	1	
>64 days	235 (40)	350 (60)	1.25 (1.06-1.47)	**0.008**	210 (36)	375 (64)	1.14 (0.97-1.33)	0.112	285 (49)	300 (51)	0.93 (0.76-1.14)	0.503

[a] GEE logistic regression clustered for multiple specimens from each infant

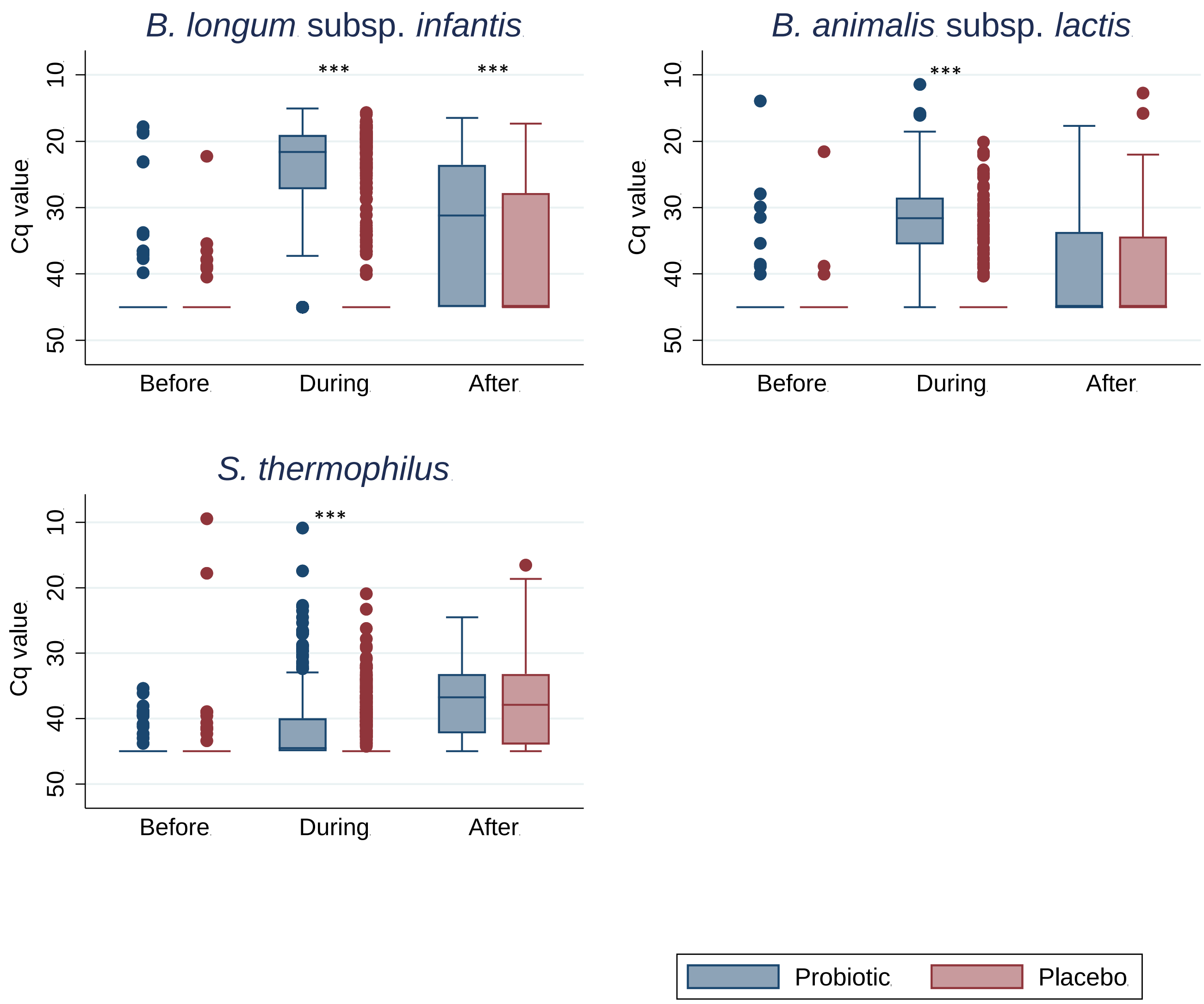

Supplementary Figure 1. Cq values of probiotic species before, during and after supplementation.
Box plots display the Cq values of *B. longum* subsp. *infantis, B. animalis* subsp. *lactis* and *S. thermophilus* stratified by randomisation group (i.e. probiotic and placebo) in specimens collected before, during and after supplementation are presented in each figure. *** indicates a significant difference ($p<0.05$) in Cq value between infants randomised to placebo vs infants randomised to probiotic as determined using linear regression fitted with generalised estimating equations.
